# Novel Indole-fused benzo-oxazepines (IFBOs) inhibit invasion of hepatocellular carcinoma by targeting IL-6 mediated JAK2/STAT3 oncogenic signals

**DOI:** 10.1038/s41598-018-24288-0

**Published:** 2018-04-12

**Authors:** Ashok K. Singh, Archana S. Bhadauria, Umesh Kumar, Vinit Raj, Amit Rai, Pranesh Kumar, Amit K. Keshari, Dinesh Kumar, Biswanath Maity, Sneha Nath, Anand Prakash, Sudipta Saha

**Affiliations:** 1 Department of Pharmaceutical Sciences, Babasaheb Bhimrao Ambedkar University, Vidya Vihar, Raibareli Road, Lucknow, 226025 India; 2 Faculty of Mathematical and Statistical Sciences, Shri Ramswaroop Memorial University, Deva Road, Lucknow, 225003 India; 3 Centre of Biomedical Research, SGPGIMS Campus, Raebareli Road, Lucknow, 226014 Uttar Pradesh India; 4 Department of Biotechnology, Babasaheb Bhimrao Ambedkar University, Vidya Vihar, Raibareli Road, Lucknow, 226025 India

## Abstract

Inspired by the well-documented tumor protecting ability of paullones, recently, we synthesized novel paullone-like scaffolds, indole-fused benzo-oxazepines (IFBOs), and screened them against hepatocellular carcinoma (HCC) specific Hep-G2 cells. Three of the synthesized compounds significantly attenuated the progression of HCC *in vitro*. By computational studies, we further discovered that IFBOs exhibited a stable binding complex with the IL-6 receptor. In this context, we investigated *in vivo* study using the nitrosodiethyl amine (NDEA)-induced HCC model, which strengthened our previous findings by showing the blockade of the IL-6 mediated JAK2/STAT3 oncogenic signaling pathway. Treatment with IFBOs showed remarkable attenuation of cellular proliferation, as evidenced through a decrease in the number of nodules, restoration of body weight, oxidative stress parameters, liver marker enzymes and histological architecture. Interestingly, using a metabolomic approach we further discovered that IFBOs can restore the perturbed metabolic profile associated with the HCC condition to normalcy. Particularly, the efficacy of compound **6a** for an anti-HCC response was significantly better than the marketed chemotherapeutic drug, 5-fluorouracil. Altogether, these remarkable findings open up possibilities of developing IFBOs as novel future candidate molecules for plausible alternatives for HCC treatment.

## Introduction

Hepatocellular carcinoma (HCC) is one of the most lethal cancers and has limited treatment options^1^. This is probably due to the fact that the precise mechanisms causing HCC pathogenicity are still unclear^2^. The response rate of sorafenib, the only FDA-approved drug for HCC therapy, is very limited despite its established efficacy^3^. Most of the sorafenib-treated patients experience disease recurrence by local metastasis and chemotherapeutic resistance^4^. Thus, the rational design of novel molecules targeting HCC specific pathways is imperative for improved therapy.

**Figure 1:**
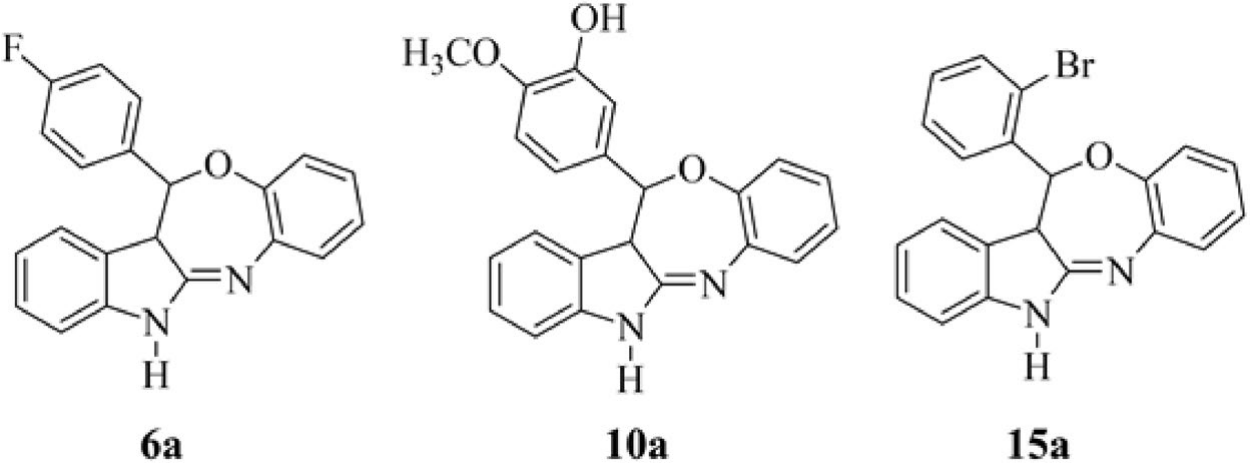
Structure of indole-fused benzo-oxazepines (IFBOs): **6a** (12-(4-Fluorophenyl)-12,12a-dihydro-5*H*-benzo[2,3][1,4]oxazepino[5,6-*b*]indole), **10a** (5-(12,12a-Dihydro-5*H*-benzo[2,3][1,4]oxazepino[5,6-*b*]indol-12-yl)-2-methoxyphenol) and **15a** (12-(2-Bromophenyl)-12,12a-dihydro-5*H*-benzo[2,3][1,4]oxazepino[5,6-*b*]indole).

The well-documented CDK1 inhibitors, “paullones,” are structurally based on indole-fused benzazepinone and can be used as biochemical tools in cancer drug discovery^5^. As evidenced through previous reports, the *in vitro* antitumor activities of a variety of paullones do not parallel with their CDK1 inhibitory properties. This finding suggests that CDKs are rather unlikely to be the critical targets responsible for the anticancer activity of paullones. Further, to potentiate the antitumor effect of paullone ring and to explore the precise mechanism of action, literatures provide two suggestions: (1) rational modifications in paullone ring structure to enhance cytotxicity, and (2) exploration of a specific target to identify the mechanism underlying paullone-modified ring^6–8^. In view of this, our research group recently reported the design and synthesis of a new paullone-like scaffold, indole-fused benzo-oxazepines (IFBOs), by rational modification of the fusion site and insertion of hetero-atoms using a computational approach^9^. The preliminary finding of *in vitro* antitumor activity against HCC specific Hep-G2 cell lines suggested that three IFBOs derivatives **6a**, **10a** and **15a** (Fig. 1) can potentially inhibit the growth of tumor cells. Again, the molecular modeling and molecular dynamic (MD) simulation study showed the formation of a stable binding complex with the interleukin-6 (IL-6) receptor molecule. In another study, we reported the pharmacokinetic properties of these three compounds (**6a**, **10a** and **15a**) with an optimal degree of oral absorption, plasma distribution and renal clearance^10^. Prior to performing in *vivo* antiproliferative study, an acute toxicity study was conducted to determine the safe dose of IFBOs in albino Wistar rats. After acquiring successful results during pharmacokinetic and acute toxicity study, we performed *in vivo* experiment to evaluate the anti-HCC action of IFBOs and to confirm the molecular mechanism underlying their action.

Growing evidences suggest that inflammation plays a major role in the initiation and progression of HCC. Epidemiological studies have confirmed that inflammatory conditions of the liver correlate well with high circulating IL-6 levels, which are even more elevated in HCC patients^11^. Excess release of IL-6 in response to inflammatory stimuli serves as a potent activator of JAK/STAT (Janus kinase-signal transducer and activator of transcription) signaling^12^. In particular, the persistent stimulation of IL-6 causes the activation of JAK2 and the subsequent phosphorylation/activation of STAT3, which contributes to tumor growth, chemo-resistance and metastasis^12,13^. Recently, a cohort based clinical study concluded that IL- 6 promotes HCC by the activation of only STAT3 rather than any other STAT members^12,14^. Further involvement of other STAT members in relation to HCC progression by various other cytokines is still unknown and beyond the scope of this study. Henceforth, we confined our study to the development of novel anti-HCC agents based on mechanistic control of the IL-6 mediated JAK2/STAT3 signaling cascade. Again, NDEA is a widely accepted carcinogen used to induce liver-specific cancer that closely resembles a subclass of human HCC^15^. Thus, the present study is vital to analyze and provide new insights into the molecular and therapeutic aspects of IL-6 mediated JAK2/STAT3 signaling, to affirm the role of the newly synthesized IFBOs in NDEA induced experimental HCC in rats.

It has recently been suggested that the carcinogenic actions of NDEA are clearly linked to oxidative stress and metabolic perturbations in liver tissue^16^. Thus, to recapitulate the characteristic features of the disease state and its cure, we also evaluated the protective effects of IFBOs against oxidative stress and profiled the systemic discriminations of plasma metabolomes between diseased and IFBOs-treated rats through ^1^H-NMR based serum metabolomics. Characteristic changes of different metabolites could offer an advanced understanding of the distinctive signature linked to NDEA-exposure and IFBOs-therapy that ultimately could inform the utility of a proof-of-principle metabolomics approach in HCC treatment.

## Results

### IFBOs exerted protection against physiological, biochemical and oxidative stresses

**Figure 2:**
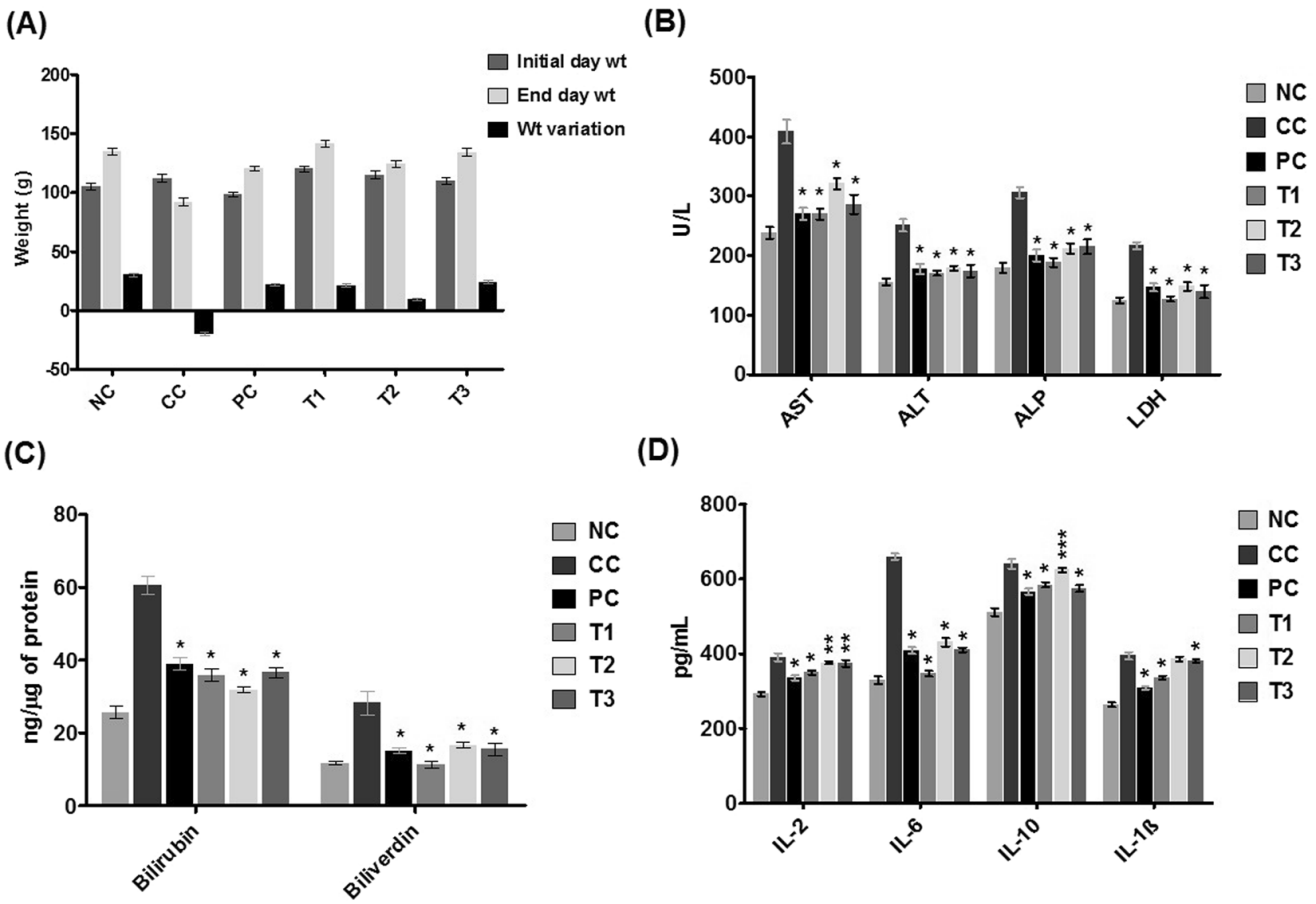
Effects of IFBOs after oral administration of 10 mg/kg for 15 days in NDEA-exposed rats **(A)** Body weight, **(B)** Enzyme levels of AST, ALT, ALP and LDH in serum, **(C)** Catabolic by-product (bilirubin and biliverdin), and **(D)** Anti-proliferative biomarkers IL-2, IL-6, IL-10 and IL-1β in liver carcinogenic tissue. Normal control (NC), Carcinogen control (CC: NDEA), Positive control (PC: NDEA + 5-FU), Treatment T1 (NDEA + **6a**), Treatment T2 (NDEA + **10a**), Treatment T3 (NDEA + **15a**). Results are expressed as mean ± SD (n = 8). Statistically significant differences were observed between carcinogen control (CC) group and test groups (PC, T1, T2 and T3). *p < 0.001, **p < 0.01 and ***p < 0.05, when compared to the carcinogen control (CC) group [one way-ANOVA followed by Bonferroni multiple comparison test].

The protective efficiency of IFBOs was assessed through various physiological parameters including body weight, percent incidence and number of carcinogenic nodules in the rat liver. The change in body weight was more prominent in the NDEA-exposed group when compared to the normal control group, whereas treatment with IFBOs successfully normalized the impact shown in the NDEA-exposed rats (Fig. 2A). Similar trends were noticed for the number of carcinogenic nodules and their % incidence; all IFBOs and 5-FU significantly lowered the NDEA-induced carcinogenic nodules by 2–6 fold, as compared to the NDEA-exposed group (Table S1, Annexure I of Supplementary material). The overall defensive effects among all IFBOs treated groups were observed in an order of T1 (NDEA + **6a**) > T2 (NDEA + **15a**) > T3 (NDEA + **10a**). Interestingly, the restoring capability shown by **6a** is more prominent and closer to the standard chemotherapeutics, 5-FU.

The antioxidant potential of the liver was investigated to correlate oxidative stress with tumor progression. Table 1 depicts the restoration of impaired oxidative stress mediators (SOD, GSH, CAT, PC and TBARS) after drug treatments. We observed that SOD and CAT enzymes were dramatically reduced in the NDEA-exposed group, whereas only a trivial reduction of these enzymes was observed for all the IFBOs and 5-FU treated groups, as compared to control. The concentration of GSH was reduced by three fold in NDEA-exposed rats; however, a significant increase (p < 0.001) in GSH concentration was observed on treatment with **6a**, **15a** and 5-FU but not with **10a**. In contrast, a notable increase in PC and MDA levels was observed in the NDEA-exposed group, but expression of these markers was most prominently regained after the treatment with all IFBOs and 5-FU.

**Table 1:** Effects of 6a, 10a and 15a treatments on oxidative stress parameters after oral administration of 10 mg/kg for 15 days.

Sr.No.	Parameters	NC	CC	PC	T1	T2	T3
1.	SOD (U/μg of protein)	0.441 ± 0.003	0.150 ± 0.010	0.400 ± 0.009*	0.356 ± 0.079*	0.383 ± 0.056*	0.329 ± 0.043*
2.	CAT (nM of H_2_O_2_/min/μg of protein)	6.06 ± 0.65	1.88 ± 0.27	5.51 ± 0.78*	5.05 ± 2.27**	5.82 ± 1.74*	5.82 ± 1.77*
3.	PC (μM/μg of protein)	0.131 ± 0.010	1.265 ± 0.050	0.186 ± 0.031*	0.165 ± 0.004*	0.168 ± 0.002*	0.164 ± 0.008*
4.	TBARS (nM of MDA/mg of protein)	38.57 ± 1.86	79.06 ± 4.73	66.20 ± 1.98*	67.73 ± 1.11*	41.08 ± 0.96*	60.82 ± 2.83*
5.	GSH (mM/mg of Protein)	17.48 ± 0.58	6.02 ± 0.77	12.04 ± 0.50*	10.26 ± 0.45*	6.50 ± 0.42	7.65 ± 0.23*

Normal control (NC), Carcinogen control (CC: NDEA), Positive control (PC: NDEA + 5-FU), Treatment T1 (NDEA + **6a**), Treatment T2 (NDEA + **10a**), Treatment T3 (NDEA + **15a**). Results are expressed as mean ± SD (n = 8). Statistically significant differences were observed between carcinogen control (CC) group and test groups (PC, T1, T2 and T3). *p < 0.001 and **p < 0.01, when compared to the carcinogen control (CC) group [one way-ANOVA followed by Bonferroni multiple comparison test].

We also analyzed the activity levels of hepatic damage markers, including AST, ALT, ALP and LDH, in the serum of the control and experimental groups of animals (Fig. 2B). Again, we noted that the NDEA-exposed group exhibited an almost two fold increase in the activity of these enzymes when compared with that of the normal control group. All treatments with IFBOs, predominantly treatment with **6a**, showed the ability of IFBOs to restore significantly the level of these markers to their near normal values.

Later, the effects of IFBOs on catabolic pigments, bilirubin and biliverdin, in hepatic tissues were also analyzed (Fig. 2C). The treatments with IFBOs reflected a significant (p < 0.001) normalization of these pigments compared with the NDEA-exposed group, which, in turn, exhibited an almost 2–3 fold elevation when compared to normal control. Further, the effects of all the IFBOs were found nearly equivalent to that of the standard drug, 5-FU.

### IFBOs efficiently reduced the level of IL-6 compared to other HCC-associated cytokines

To explore the effects of newly synthesized IFBOs on inflammatory events, we performed enzyme linked immunosorbent assays (ELISA) to investigate the concentrations of HCC-associated cytokines, IL-2, IL-6, IL-1β and IL-10, in rat liver (Fig. 2D). The concentrations of all these molecular markers were dramatically increased in the liver of the NDEA-exposed group compared to normal control, with a higher increase observed for IL-6 than the other cytokines. All the tested IFBOs efficiently suppressed the abnormally high levels of these mediators in liver tissues. Although the treatment with IFBOs attenuated the increased levels of all the cytokines (IL-1β, IL-2, IL-6, and IL-10), the effect was approximately 2-fold more pronounced for the inhibition of IL-6 as compared to the other cytokines. In addition, the group treated with compound **6a** showed a more significant inhibition (p < 0.001) of IL-6 than the other IFBOs and 5-FU as well.

### IFBO treatment restored the liver morphology in NDEA-exposed rats

**Figure 3:**
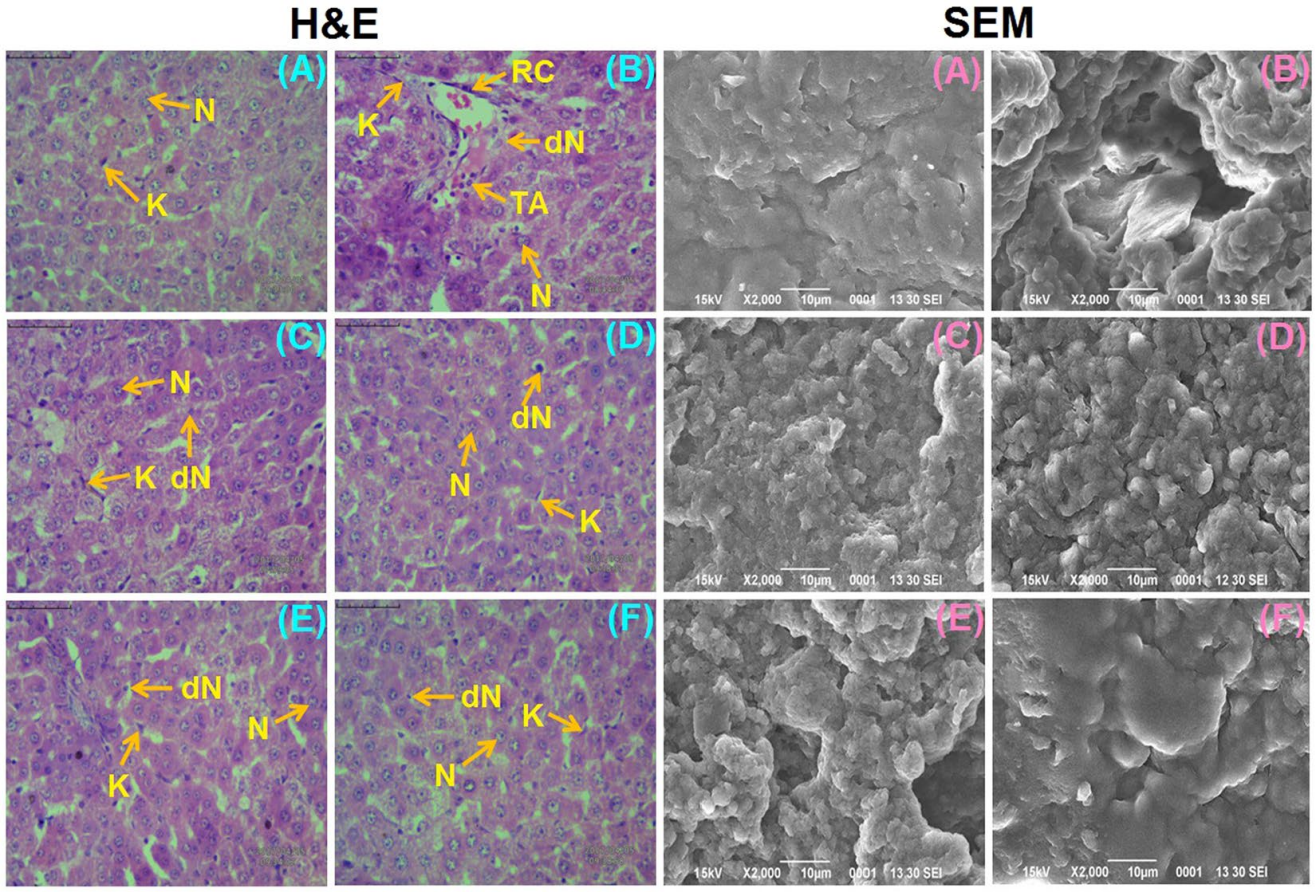
The histopathological changes (40 × , Scale bar 50 µm) and scanning electron microscopy (X2000) of the liver tissue samples in NDEA-induced HCC rats (**A**) Normal control (NC), (B) Carcinogen control (CC: NDEA), (**C**) Positive control (PC: NDEA + 5-FU), (**D**) T1 (NDEA + **6a**), (E) T2 (NDEA + **10a**) and (F) T3 (NDEA + **15a**). Normal nucleus (N), degenerated nucleus (dN), ruptured hepatic cells (RC), Tumor anaplastic cells (TA), Kupffer cells (K). Induction of carcinogenesis by NDEA is clearly visualized in the diseased group (**B**). Treated groups (**C**–**F**) images show the restoration of liver cell architecture by IFBOs.

Histological changes in liver tissue were noticed in various treated or control groups by hematoxylin and eosin (H & E) staining (Fig. 3). In normal control animals, the sections showed normal architecture of cells with nuclei, whereas the liver of NDEA-exposed animals exhibited an architecture disturbance and the presence of binucleate, enlarged, polygonal hepatocytes with acidophilus staining in the cytoplasm. In particular, the liver sections of diseased animals exhibited irregular sinusoids, tumor anaplastic cells (TA), degenerated nuclei (dN), ruptured hepatic cells (RC) or Kupffer cells (K) and tumoral vacuoles. There was a marked improvement in the gross microscopic appearance of liver tissue, when IFBOs at 10 mg/kg were supplemented to NDEA-exposed animals. SEM analysis (Fig. 3) also showed similar patterns, where lesions were less prominent in IFBO treated groups as compared to the NDEA-exposed group, and the effects were almost similar to that of the group treated with 5-FU.

### IFBOs down-regulated mRNA expression of IL-6

**Figure 4:**
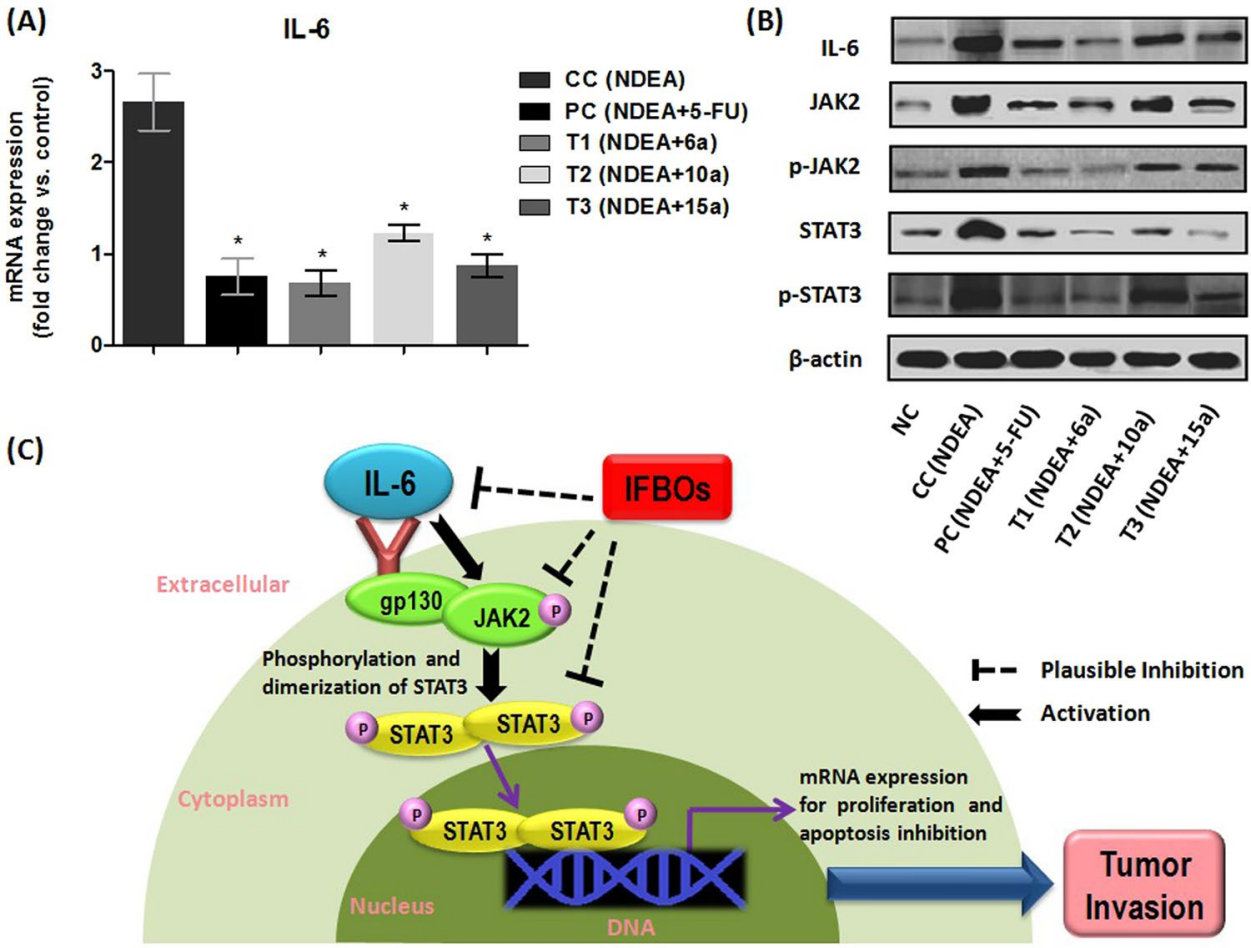
**(A)** mRNA expression levels of IL-6. qRT-PCR analysis confirms IFBOs potential to regulate the expression of IL-6. Results are expressed as mean ± SD (n = 8). Statistically significant differences were observed between carcinogen control (CC) group and test groups (PC, T1, T2 and T3). *p < 0.001, when compared to the carcinogen control (CC) group [Paired T-test] **(B)** Protein expression levels of IL-6, JAK2, p-JAK2, STAT3 and p-STAT3. Immunoblot analysis confirms IFBOs potential to regulate the expression of IL-6/JAK2/STAT3 signaling. (**C**) The plausible antitumor activity of IFBOs by the inhibition of IL-6-mediated JAK2/STAT3 activation: Firstly, IL-6 is released strongly in response to various inflammatory stimuli. IL-6 serves as potent JAK2/STAT3 activator by inducing the tyrosine phosphorylation (P) of JAK2 and STAT3. The activated p-STAT3 forms the homo- or heterodimers, which translocate to nucleus, bind to DNA and transcript several oncogenes to accelerate the tumor progression. IFBOs can inhibit the over-expression of IL-6 and IL-6 mediated JAK2 and STAT3.

We already confirmed that IFBOs provide comparatively more impact on the IL-6 level than any other tested cytokine. Thus, we hypothesized that the anti-HCC action of IFBOs might be due to the down-regulation of the over-expressed IL-6 gene. In order to explore the underlying mechanism, qRT-PCR analysis was performed to investigate if IFBOs have a capacity to regulate the elevated mRNA expression of IL-6 in the HCC condition. Our findings suggested that the IL-6 level was dramatically elevated in the NDEA-exposed group as compared to that of the normal control group. However, treatment with IFBOs significantly normalized (p < 0.001) the over-expression of the IL-6 gene. The efficacy of the IFBOs at a 10 mg/kg dose was comparable to the market available chemotherapeutics 5-FU at a 10 mg/kg dose, and compound **6a** was found to be somewhat more potent than the standard chemotherapeutic drug, 5-FU. (Fig. 4A).

### IFBOs attenuated the protein expression of IL-6, p-JAK2, STAT3 and p-STAT3

To check the protein expression levels of IL-6 and JAK2/STAT3, we performed immunoblot analysis of the liver tissues of the experimental groups (Fig. 4B). The expression level of IL-6 was elevated in the NDEA-exposed group and decreased in all three IFBO treated groups. In particular, a marked obliteration in IL-6 expression appeared for the **6a** treated group, and the expression was found to be very close to normalcy. Further, JAK2, p-JAK2, STAT3 and p-STAT3 showed increased expression in NDEA-exposed, diseased group, whereas a moderate to excellent attenuation of their expression levels was found in different IFBOs treated groups. The obliteration in p-JAK2, p-STAT3 and STAT3 expression levels was more pronounced in the **6a** treated group. Again, the **15a** treated group also showed a marked reduction in STAT3 expression, but the effect of **10a** was somewhat less as compared to those of **6a** and **15a**. Interestingly, the effects of **6a** on IL-6, p-JAK2, p-STAT3 and STAT3 expression were comparable or somewhat better than the positive control, 5-FU. The possible mechanistic role of the IFBOs towards the IL-6 mediated JAK2/STAT3 signaling blockade to protect against tumor invasion is represented in Fig. 4C.

### IFBOs restored the serum metabolite profiles in NDEA-exposed rats

**Figure 5:**
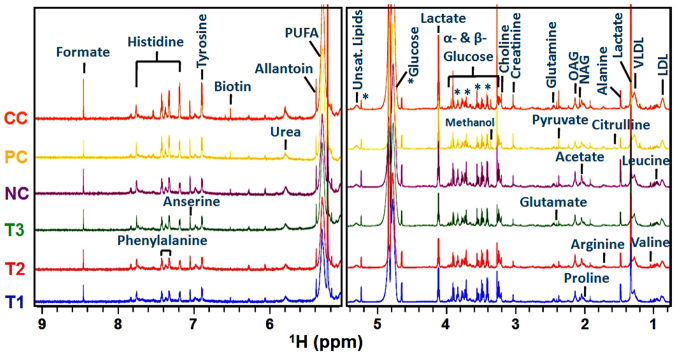
Stack plot of representative 1D ^1^H CPMG NMR spectra of rat serum obtained from different groups. Normal control (NC), Carcinogen control (CC: NDEA), Positive control (PC: NDEA + 5-FU), T1 (NDEA + **6a**), T2 (NDEA + **10a**), T3 (NDEA + **15a**). The abbreviations used are: LDL/VLDL: Low/very-low density lipoproteins; N-acetylglycoprotein: NAG, *O*-acetyl glycoprotein: OAG; Unsat. Lipids: Unsaturated lipids.

**Figure 6:**
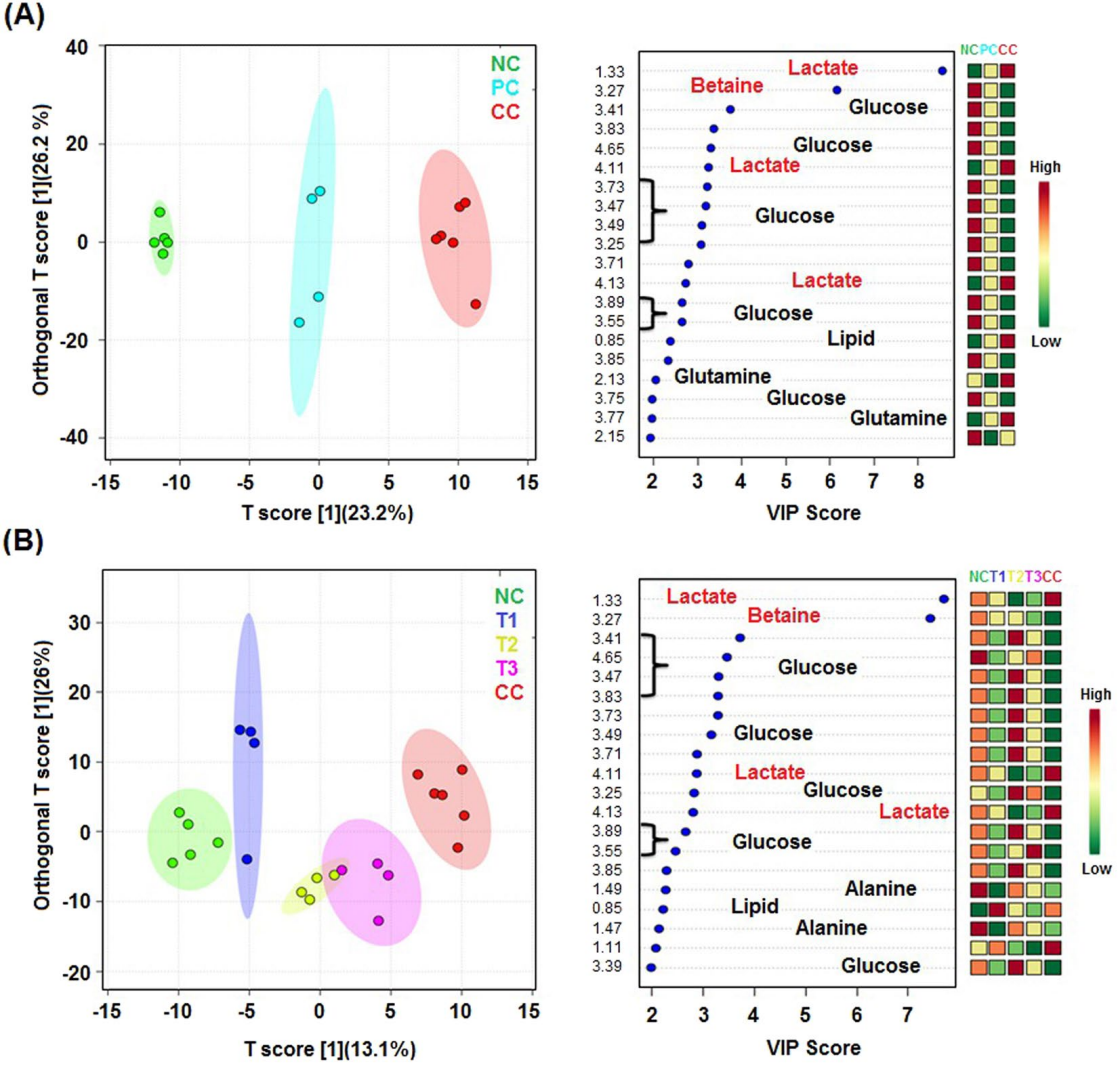
Combined and pair-wise 2D OPLS-DA analysis of 1D ^1^H CPMG NMR spectra score plot **(A)** for the groups: NC (Normal control), CC (Carcinogen control: NDEA), PC (Positive control: NDEA + 5-FU), and **(B)** for the groups: NC (Normal control), T1 (NDEA + **6a**), T2 (NDEA + **10a**), T3 (NDEA + **15a**), CC (Carcinogen control: NDEA). The potential discriminatory metabolites identified from VIP scores are derived from PLS-DA modeling of complete data matrix, and resulted VIP scores for top 20 metabolites are shown in increasing order of VIP score values to highlight their discriminatory potential.

**Figure 7:**
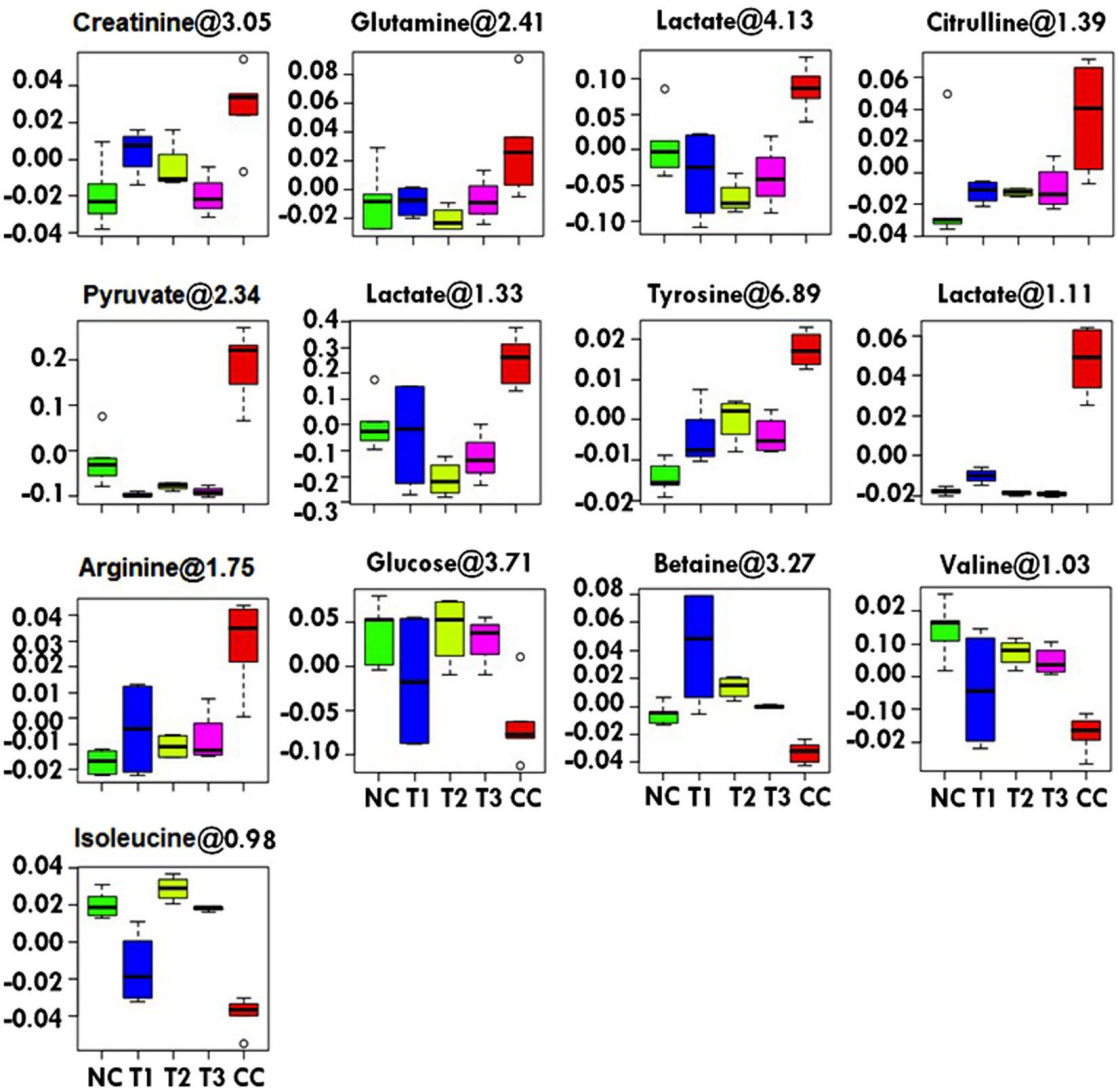
Metabolic effects of **6a**, **10a** and **15a** treatments: The box-cum-whisker plots are showing relative variations in quantitative profiles of serum metabolites relevant in the context of the pathophysiology of liver cancer. In the box plots, the boxes denote interquartile ranges, horizontal line inside the box denote the median, and bottom and top boundaries of boxes are 25th and 75th percentiles, respectively. Lower and upper whiskers are 5th and 95th percentiles, respectively. NC (Normal control), CC (Carcinogen control: NDEA), T1 (NDEA + **6a**), T2 (NDEA + **10a**) and T3 (NDEA + **15a**).

The NMR data of the serum samples of different studied groups were evaluated using SIMCA-P software, version 11.0 (Umetrics AB, Umea, Sweden) for which multivariate analyses such as principal component analysis (PCA) and orthogonal partial least squares-discriminant analysis (OPLS-DA) were executed to monitor the metabolic perturbations in a supervised and unsupervised manner, respectively. To validate the analytical quality of system performance and to observe the plausible outliers, a PCA model was employed. The OPLS-DA model was found beneficial to acquire a summary of the complete data set of different samples and to obtain a discriminative signature responsible for changes among all the studied groups. The quality of the OPLS-DA model was authenticated with two variables, i.e. Q^2^ and R^2^Y. The score plots acquired from 1D ^1^H CPMG NMR spectra (Fig. 5) displayed reasonable separation among the studied groups (Fig. 6). The selection of various metabolites was based on the statistically significant threshold of variable influence on projection (VIP) values greater than 1.0. Simultaneously, the *p*-value < 0.05 from Student’s t-test on the regulated peak area demonstrated statistically significant. Log2 fold change (FC) was applied to reveal the particular metabolites discriminations among all the studied groups. The datasets of these metabolic discriminations (log2-scaled) were imported into MetaboloAnalyst 3.0 for heat maps generation and multivariate statistical analysis. The receiver operating characteristic curves (ROC) were used to evaluate the potential of effective biomarkers. The statistically significant data were considered at *p* $<$ 0.05 (Fig. 7).

Considering the PLS-DA data, the combined as well as the pair wise analysis displayed R^2^ = 0.70 and Q^2^Y ≥ 0.90, which indicated a significant metabolic difference between the NDEA-exposed group and the IFBO treated groups. The key metabolic perturbations between the normal control and NDEA-exposed groups, along with their chemical shifts (δ) values, variable importance on projection (VIP) score and p-value, are depicted in Figs 5 and 7.

Typical ^1^H CPMG NMR spectra of serum samples of various studied groups are shown in Fig. 5. The NMR spectra showed signals, mainly from lipids/lipoproteins [(e.g., low density lipoprotein (LDL), very low density lipoprotein (VLDL), polyunsaturated fatty acid (PUFA), and unsaturated lipids)], membrane metabolites (e.g., choline), N-acetyl and *O*-acetyl glycoproteins (NAG, OAG), and amino acids (e.g., arginine, valine, isoleucine, alanine, proline, glutamine, histidine, glutamate, tyrosine, and phenylalanine). Other identified metabolites were glucose, lactate, pyruvate, acetate, formate, citrulline, creatinine, urea, biotin, anserine and allantoin.

Representative box-cum-whisker plots, presented in Fig. 7, depict the significant perturbations in the profile of these metabolites. A significant elevation in the levels of pyruvate, lactate, glutamine, citrulline, creatinine, tyrosine, and arginine was observed in NDEA-exposed rats. Moreover, a significant decrease in the levels of betaine, glucose, valine and isoleucine were recorded in NDEA treated rats. All of these metabolic alterations were successfully retrieved with the administration of IFBOs, as evident from the box plots.

## Discussion

Paullones, chemically indole-fused benzazepinones, are described as potent inhibitors of cyclin-dependent kinases (CDKs) from the data analysis of an anti-cancer drug screen of the National Cancer Institute’s tumor cell lines^17,18^ and have drawn major research interests for anti-cancer drug discovery. However, many controversies still exist regarding the association between the antiproliferative action and CDK inhibitory activities of paullones. This is evidenced by the fact that the CDK inhibitory activity (IC_50_) of paullones seems to occur regardless of their respective antiproliferative activity (GI_50_) on a panel of tumor cells^6–8^. In view of these problems associated with the paullone ring, literatures have suggested that there probably remains a great opportunity for improving the efficacy of paullones by modifying the paullone skeleton or with the exploration of actual targets other than CDKs^6–8,19^. Thus, our interest lies not only in the development of a paullone-like skeleton but also to identify more appropriate targets other than CDKs. Henceforth, recently, we designed a new pharmacophoric scaffold, phenyl substituted indole-fused benzo-oxazepines (IFBOs) by the rational modification of the fusion site and insertion of a hetero atom in the paullone skeleton. Among them, three IFBO derived compounds, **6a**, **10a** and **15a**, displayed excellent cytotoxicity on Hep-G2 cells and formed a stable binding complex with the IL-6 receptor^9^. Our current study focused on the *in vivo* anti-tumor effect of IFBOs in the NDEA-induced HCC model and assembled a few notable findings exploiting the directed approach to prove the mechanisms underlying IFBO action.

In our preliminary investigations, reduced body weight and higher incidence of carcinogenic nodules indicated carcinogenic condition in NDEA-exposed rats. The observed reduction in these changes after IFBO (10 mg/kg) administration was primary indication of its protective action in NDEA induced HCC model, which suggested further requirement for biochemical and pathophysiological analysis. It has previously been documented that the activities of antioxidants decreases during HCC conditions^20^. Reactive oxygen species (ROS) are generally produced in all aerobic cells and counter balanced by the antioxidant enzymatic defense^21^. However, this counter balance effects are attenuated during anaerobic/hypoxic conditions like cancer (due to excessive cellular proliferation)^22^. In fact, the increased ROS production inhibits the enzymatic antioxidant defense of GSH, SOD and CAT, as they all work together to curtail ROS through a series of peroxidation, dismutation and oxidation reactions^23^. The decrease in the enzymatic defense of GSH, SOD and CAT, suggest their increased utilization during excessive cellular proliferation, which was profoundly evident after the NDEA treatment. Further, the damage to the cellular lipids and proteins can be validated through increased production of MDA and PC respectively^24^; which was very well evident after the NDEA treatment. Deregulation of these parameters suggested the onset of oxidative stress condition that leads to carcinogenesis through impaired intracellular signaling pathways with altered expression of oncogenes^25^. It would be appropriate to note that IFBO administration curtailed the levels of MDA and PC with restoration of enzymatic antioxidant defense of GSH, SOD and CAT, substantiating its tumor protecting ability with notable antioxidant effects.

Further, the elevated levels of catabolic by-products bilirubin and biliverdin indicate hepatic disease^26^. The elevation in these markers during NDEA exposure and their normalization through IFBOs treatment also supported the hepatic disease control. Moreover, elevation of AST and ALT levels in serum are indications of cirrhosis and hepatic liver injury^27^. Among liver function tests, ALP is an independent factor for HCC patient survival, and its preoperative level can be utilized to monitor recurrence in high risk HCC patients^28^. Henceforth, the elevated levels observed for the liver function enzymes (AST, ALT and ALP) reflect the advancement of carcinogenesis in diseased animals. The observed efficacy of IFBOs to restore these enzyme levels indicated the ability of IFBOs to prevent hepatic damage^12^. Similarly, a noteworthy increase in serum LDH level shows a nonspecific alteration in the cell membrane integrity and liver metastases of HCC^29^. Thus, the high levels of serum LDH obtained in the NDEA-exposed group could be responsible for NDEA mediated liver damage and the occurrence of pre-neoplastic lesions. Nevertheless, IFBO supplementation led to the abatement of hepatic damage and alleviation of pre-neoplastic lesions, probably by its capacity to improve LDH enzyme levels and thereby potentiate its anti-tumor activity.

Further evidence of protective action was observed through histopathology and SEM analysis. Both analyses showed that the NDEA-treated animals exhibited irregular cytoplasm with irregular shaped nuclei, probably due to excessive free radical generation during NDEA exposure^24^. The less ruptured and denatured cells (RC and dN) in IFBO-treated groups, compared to NDEA-exposed group, signified the ameliorative potential of IFBOs against HCC.

Serum cytokines are key regulators for several pathological and physiological alterations involving inflammation and cancer invasion. Recent reports suggested that most of the pro-inflammatory cytokines, including IL-2, IL-6, IL-1β and IL-10, are over-expressed at cancer specific sites and particularly linked to invasion and progression of sever HCC conditions^30^. According to other reports, the enhanced expression and formation of cytokines can mediate cascade amplification of the inflammatory response and liver cell damage which can actively contribute to cancer initiation and progression in liver tissue^30–33^. Consequently, we investigated the altered levels of these mediators in the NDEA-exposed group and the effects of IFBO treatment on them through ELISA. As the result, the most efficient restoration of IL-6 levels by IFBOs provided evidence of their cellular functioning through the curtailment of IL-6 gene over-expression at cancer sites. This evidence is corroborated well with our previous literature^9^ where MD simulation led to the formation of a stable binding complex between IFBOs and the IL-6 receptor.

Further, over-expression of IL-6 gene has been clearly linked to elevated cancer risk and HCC prognosis^33^. In qRT-PCR analysis, the rapid reduction in over-expressed mRNA level of IL-6 through IFBO treatment provided a trend similar to those measured by ELISA. Furthermore, it is well documented that IL-6 is released strongly in response to various inflammatory stimuli and serves as a potent JAK/STAT activator. In particular, the HCC-causing oncogenic effect is mediated through persistent stimulation of IL-6, leading to the phosphorylation/activation of JAK2 followed by phosphorylation/activation of STAT3. This activated STAT3 undergoes homo or hetero-dimerization that leads to nuclear translocation, DNA binding and subsequently gene transcription of several oncogenes involved in initiation and progression of HCC^13,34,35^. From the results of western blotting, we confirmed that IFBOs could manifest its effects through blockade of the IL-6/JAK2/STAT3 signaling cascade in NDEA-induced carcinogenic rats. Firstly, IFBO treatment favored the curtailment of the IL-6 protein expression level which substantiated the qRT-PCR study where decreased mRNA expression of IL-6 was observed. Secondly, IFBO treatment favored the decreased expression of p-JAK2 and p-STAT3 with concomitant reduction in STAT3 expression. Thus, it would be appropriate to state that IFBO treatment can produce not only inhibition of JAK2/STAT3 phosphorylation but also the direct inhibition of STAT3. Thus, herein we report new *in vivo* findings of our newly reported IFBOs that manifest an anti-tumor therapeutic effect by the favorable regulation of the IL-6-JAK2-STAT3 signaling nexus during NDEA-induced HCC condition. The insights provided in this study depicts a new molecular mechanism of action of the totally novel paullone-like ring, IFBO, that completely differs from the expected mechanism of antiproliferative action of well-documented paullones.

We further implemented ^1^H NMR based metabolomics to evaluate whether IFBOs have the ability to restore the metabolic perturbations during the NDEA-exposed HCC condition. OPLS-DA score plots and box-cum-whisker plots using MetaboAnalyst^36^ were obtained from 1D ^1^H CPMG NMR spectral data and revealed significant metabolic alterations in the cancerous condition. Interestingly, a decreased glucose level and increased lactate level were observed in the NDEA-exposed group, which was well corroborated by earlier findings that demonstrate the cancerous condition^37,38^. The result obtained in this study supported the Warburg effect that could be associated to a higher amount of glucose consumption in cancer condition, followed by the formation of lactate as a by-product^39,40^. The notable event recorded in this study demonstrated the excellent ability of IFBOs to restore two major metabolic perturbations, i.e., reduced glucose and elevated lactate levels, a hallmark for tumor progression. Similarly, a significant elevation in lipoproteins and lipids in HCC rats was noted, when compared to normal control rats. These lipoproteins are used to transport the hydrophobic lipid molecules in circulation and also involved in energy production, for example, through β-oxidation. Thus, the elevated level of lipoproteins and lipids could be the consequence of the energy requirement for the synthesis of cell membrane and rapid proliferation^41^. Moreover, arginine is critical for the growth of human cancers owing to its active involvement in tumor metabolism, including protein synthesis as well as synthesis of polyamines, nitric oxide, glutamate, proline and nucleotides^42^. An elevation in arginine level in the NDEA-exposed group suggests its increased requirement to synthesize key molecules in rapidly proliferating tumor cells. Furthermore, serum creatinine, a key intermediate in energy metabolism, was considerably increased in NDEA-exposed HCC rats and could be linked to a higher energy demand for cancer cell proliferation. Tyrosine was also up-regulated in the NDEA-exposed group that can be an indication of enhanced catabolism during the cancerous condition^37,43^. The elevated levels of pyruvate and glutamine were corroborated well with a previous report on human HCC^44^. We found that NDEA-induced HCC sera showed higher levels of pyruvate compared to a normal individual. The increased level of pyruvate in HCC serum is possibly due to the decreased utilization of pyruvate in the TCA cycle or an increase in anaerobic cell respiration. Similarly, we noticed a higher concentration of glutamine in NDEA-induced HCC sera compared to normal individuals. The elevated level of glutamine in the HCC condition could be a result of the accumulation of α-ketoglutarate, which is fluxed out of the mitochondria and converted into glutamine in the cytosol. Moreover, in accordance with Raouf *et al*.^45^, our study showed a significant increase in the citrulline level, in the HCC condition. Betaine is mainly involved in choline metabolism and down-regulated in the NDEA-exposed group, further providing evidence of excessive cellular proliferation^37^. The depleted levels of branched-chain amino acids (BCAA) valine and isoleucine were in accordance with the previous report on human HCC^44^. With all the above metabolic modulations and their regulations by IFBOs, it may be concluded that our study is well corroborated with earlier reported principal metabolic alternations showing elevated glycolysis or gluconeogenesis (glucose and lactate), and β-oxidation (lipoproteins and lipids) with reduced tricarboxylic acid (TCA) cycle (pyruvate, glutamine) utilization during the HCC condition^37^. Almost all the aforementioned metabolic changes in NDEA-exposed group were reset back to normal after IFBO administration, suggesting that IFBOs have potential to balance the metabolic abnormalities in rapidly dividing tumor cells.

In conclusion, we herein employed a short rational method to discover the molecular mechanism of our newly synthesized paullone-modified skeleton, IFBO. The current study substantiates the biochemical, pathophysiological and molecular link of IFBO treatment and demonstrates the mechanism of their anti-tumor activity. Our *in vivo* result confirms those of the previous *in vitro* study. The IL-6 mediated JAK2-STAT3 inhibitory properties of all the three IFBOs was parallel to their respective *in vitro* antitumor activity, showing an almost similar order of activity in all cases, i.e., **6a** > **15a** > **10a**. Although the molecular insights discovered for IFBO action renders the blockade of a well-known IL-6-JAK2-STAT3 signaling pathway, it completely differs from the controversial cytotoxic mechanisms of paullones. Moreover, using a metabolomics approach in an *in vivo* model, we discovered an advanced mechanistic understanding of IFBO action at cellular level. Taken together, all the results indicate that IFBOs show exceptional potential to obliterate HCC and could serve as potential lead molecules for the development of anti-HCC drugs.

## Materials and Methods

### Drugs and Reagents

The three IFBO derivatives, **6a**, **10a** and **15a** were already synthesized by our research group^9^. Other required chemicals, like GSH, NDEA and 2,4-dinitrophenylhydrazine (DNPH), were procured from Sigma-Aldrich, USA. AST, ALT, ALP and LDH kits were acquired from the Transasia Biomedicals Pvt. Ltd., Baddi, India. Two interleukins (IL-2 and IL-6) were commercially purchased from Sigma-Aldrich, USA whereas other interleukins (IL-1β and IL-10) were obtained from Genetex Biotech Asia Pvt. Ltd, New Delhi, India. All the antibodies were procured from Thermo Fisher Scientific, Waltham, MA, USA. All solvents and chemicals were of analytical reagent grade unless otherwise stated, and whole experiment was carried out using doubly distilled water.

### Experimental animals

Healthy male albino Wistar rats of weight ranging 80–120 g, were used for the experiment after the prior approval of the protocol by the Institutional Animal Ethical Committee (IAEC) (Ref. No. SDCOP&VS/AH/CPCSEA/01/0038/R3). All methods were followed in accordance with the relevant guidelines and regulations of IAEC. Standard laboratory conditions (at 25 ± 5 °C temperature; 44–56% relative humidity; 12 h light:dark cycle) were provided to acclimatize the animals with free access to water, *ad libitum* and standard rat chow. Animals were kept for one week before starting the experiment.

### Acute toxicity study

An acute toxicity study with the IFBOs was conducted according to the revised guidelines 423 of Organization for Economic Cooperation and Development (OECD). Test compounds were dissolved in 0.25% carboxymethyl cellulose (CMC) solution and given orally at the doses of 5 and 10 mg/kg to albino Wistar rats for 15 days (n = 10). All the animals were observed every day for any toxic manifestations. Neither any toxic manifestations nor behavioral changes were observed post 15 days treatment and all the three IFBOs were confirmed safe up to a dose of 10 mg/kg (Data not shown). We therefore decided to assess *in vivo* activity against HCC at a dose of 10 mg/kg body weight.

### Experimental design

Six-week-old albino Wistar rats were allocated randomly into 6 groups with 8 animals in each group (n = 8), and the classification of the groups was as follows: Group 1 (Normal Control group): 0.25% CMC (2 mL/kg), Group 2 (Carcinogen Control/NDEA-exposed group): NDEA (100 mg/kg, intraperitoneal. once a week for 6 weeks)^46–48^, Group 3 (Positive Control group): NDEA + 5-FU (10 mg/kg, intraperitoneal. for 15 days treatment after the induction of HCC), Group 4: T1 (NDEA + **6a)** (10 mg/kg, orally for 15 days treatment after the induction of HCC), Group 5: T2 (NDEA + **10a)** (10 mg/kg, orally for 15 days treatment after the induction of HCC), and Group 6: T3 (NDEA + **15a)** (10 mg/kg, orally for 15 days treatment after the induction of HCC).

Recent reports^47,48^ have clearly mentioned that HCC is induced in Wistar albino rats by i.p. injection of NDEA at a dose of 100 mg/kg once a week for 6 weeks. By adopting this protocol, NDEA was administrated to all animals of groups 2 to 6 after acclimatization for one week initially. After the 6 week completion of NDEA administration, 5-FU and the three IFBOs (**6a**, **10a** and **15a**) were given to the animals of group 3, 4, 5 and 6, respectively, for 15 days. All the animals were sacrificed by cervical decapitation after the completion of the treatment, and the livers were dissected out promptly, rinsed in ice-cold saline and kept at −80 °C for further morphological studies, oxidative parameters analysis and to explore gene as well as protein expression at the molecular level. The rat serum was collected and stored for further bio-chemical analysis and to elucidate ^1^H-NMR based metabolite profiling.

### Estimation of physiological parameters

The initial and final body weight during the experiment was measured to calculate the % weight gain. The number of carcinogenic nodules, and their % incidence was also determined to monitor the cytotoxicity profile of treated and untreated groups.

### Estimation of various enzymes and antioxidant markers

The enzyme levels in the serum, including aspartate aminotransferase (AST), alanine aminotransferase (ALT), alkaline phosphatase (ALP)^49^ and lactate dehydrogenase (LDH), were estimated using kits available commercially. The oxidative stress parameters, including superoxide dismutase (SOD), catalase (CAT), glutathione (GSH), protein carbonyl (PC), and thiobarbituric acid reactive substances (TBARS)^50^, were estimated in liver tissue of rats. The procedures adopted for the estimation of these markers are described in Annexure II of the Supplementary material.

### Estimation of catabolic by-products

The procedures adopted for determination of catabolic by-products (bilirubin and biliverdin)^50^ in liver tissue are described in Annexure III of the Supplementary material.

### Histopathological and SEM analysis of liver tissue

The procedures followed for histopathology and SEM^50^ are described in Annexure IV of the Supplementary material.

### Estimation of cytokines by ELISA

Altered levels of pro-inflammatory cytokines, including different interleukins IL-1β, IL-2, IL-6 and IL-10 in liver tissue were examined by ELISA as per the manufacturer instructions.

### qRT-PCR analysis

To explore the mRNA expression levels for the target gene, 10 mg tissue sample was taken from each group, and the isolation of total mRNA was carried out using the TriZol reagent. The RNeasy mini kit was applied for the purification of the mRNA. The manufacturer’s protocol for GeneSure cDNA synthesis kit (Genetix Biotech Asia Pvt. Ltd., New Delhi, India), was employed to prepare cDNA. Finally, qRT-PCR was performed by an Agilent Stratagene Mx3000P series (Applied Biosystems, Foster City, USA) with the help of the Sybr^@^ green PCR master mix. The cDNA was denatured at 94 °C for 5 min, annealed at 58 °C for 30 s and further elongation was performed at 72 °C for 35 s. Forty times repetition of the cycle was set using qRT-PCR which helps in detection of the amplified DNA in real time. The housekeeping control GAPDH was used to normalize mRNA. For all the treated groups, ΔCt values were normalized with the help of the untreated control samples (ΔCt = Ct _gene of interest_ − Ct _housekeeping gene_). Relative changes in the expression level of a particular gene were measured in terms of 2^−ΔΔCt^ (ΔΔCt = ΔCt _test_ − ΔCt _control_)^51^. The primer sequences were taken as follows: GAPDH, 5′-TGATGGGTTTCCCATTGATGA-3′ (forward) and 5′-TGATTCTACCCACGGCAAGTT-3′ (reverse); IL-6, 5′-TCAATGAGGAGACTTGCCTG-3′ (forward), 5′-GATGAGTTGTCATGTCCTGC-3′ (reverse)^52^.

### Protein expression by western blot

Protein expression levels of IL-6, JAK2, p-JAK2, STAT3, p-STAT3 and β-actin were assessed by immunoblotting. Cells were lysed in RIPA buffer, centrifuged at 10,000 rpm for 15 min at 4 °C, and protein amounts were quantified using Bradford’s reagent. Proteins were electrophoresed on 12% SDS-polyacrylamide gels and immediately transferred to polyvinylidene fluoride membranes. The membranes were blocked in 5% skimmed milk containing 0.1% Tween-20 in PBS for 3 h at 4 °C. Then, the membranes were incubated at 4 °C overnight with the primary antibody: IL-6, JAK2, p-JAK2, STAT3, p-STAT3 and β-actin. The next day, the membranes were washed with tris-buffered saline containing tween-20 (TBST) three times and incubated with the secondary antibody (conjugated horseradish peroxidase) at room temperature for 3 h. The film was washed three times with TBST. Then, the membrane was developed with ECL (Pierce™ ECL Western Blotting Substrate), and the images were derived from Chemidoc (Clinx Scientific Instruments, China). β-actin was used as an endogenous control^53,54^.

### ^1^H-NMR based serum metabolomics

Sample preparation and ^1^H-NMR processing: A mixture of 250 μL of serum from each sample and 250 µL of 0.9% saline sodium-phosphate buffer (50 mM, pH 7.4 in 100% D_2_O) were taken in 2 mL Eppendorf tubes, mixed well via vortex mixer and centrifuged at 10,000 rpm for 5 min. The supernatant (400 µL) was transferred to 5 mm NMR tubes (Wilmad Glass, USA) for data acquisition. 0.1% TSP (Sodium salt of 3-trimethylsilyl-(2,2,3,3-d4)-propionic acid) as an external standard, was mixed in an NMR tube to aid metabolite quantification.

The NMR spectra of the finally prepared samples were recorded at 298 K with the help of Bruker Biospin Avance-III 800 MHz NMR spectrometer, fixed at a proton frequency of 800.21 MHz. The NMR spectrometer was equipped with the CryoProbe shielded maximum gradient-strength output of 53 G/cm. The 400 µL of serum sample was filled in 5 mm NMR sealed tube and a sealed capillary tube containing the known concentration of TSP was inserted separately for the purpose of locking and chemical shift referencing. For each serum sample, transverse relaxation-edited CPMG (Carr–Purcell–Meiboom–Gill) NMR spectra were acquired using the standard Bruker’s pulse program library sequence (CPMGPR1D) with pre-saturation of the water peak by irradiating it continuously during the recycle delay (RD) of 5 s. Each CPMG NMR spectrum was consisted of the accumulation of 128 scans and lasted for 15 minutes approximately. A total spin–spin relaxation time of 60 ms (n = 300 and 2τ = 200 δs) was employed to remove all the broad signals of proteins, triglycerides, phospholipids and cholesterols. Again, a diffusion time of 120 ms was employed to attenuate the signals of low molecular weight compounds without affecting the lipid signals. All the raw NMR data were acquired using Topspin-2.1 (Bruker NMR data Processing Software) followed by the application of standard Fourier Transformation (FT) procedure for phase and baseline correction. Prior to FT, each free induction decay (FID) was filled to zero and Fourier-transform (FT) was adjusted to 4096 data points with the application of sine-bell apodization function/tapering function. After FT, the reference peak was adjusted internally to the methyl doublet of L-lactate (δ = 1.33 ppm). All the recorded spectra were visually monitored for acceptability and subjected to multivariate statistical analysis to distinguish the perturbed metabolic pattern.

### Spectral Assignment

To assign the different peaks in the 1D ^1^H-CPMG NMR spectra, two-dimensional NMR (2D NMR) spectra were recorded for selected samples, including total correlation spectroscopy (TOCSY) and heteronuclear single quantum correlation (HSQC). The identification and assignment of the chemical shifts were conducted by comparing the chemical shifts with the the database library of Chenomx 8.1 software NMR suite (Chenomx Inc., Edmonton, Canada). The remaining peaks in the CPMG NMR spectra were allocated by adopting the previously reported NMR databases of metabolites obtained from the HMDB (The Human Metabolome Database) and BMRB (Biological Magnetic Resonance Data Bank)^55,56^.

### Multivariate data analysis

The multivariate data analysis was conducted using ^1^H-CPMG NMR spectra. All the ^1^H-NMR spectra were manually corrected for phase and baseline aberration using Topspin 2.1 (Bruker NMR data Processing Software). For multivariate analysis, the CPMG spectra (at δ 8.5–0.5 ppm) were binned into 0.01 ppm wide integrated spectral buckets and integrated automatically using AMIX package (Version 3.8.7, Bruker, Bio Spin). ^1^H-CPMG spectra have signals both from low molecular weight (MW) metabolites and lipid metabolites. It is notable that the ^1^H-CPMG NMR is generally used by suppressing the wide peaks to quantify the small molecular weight metabolites. The quantitative difference of lipid signals may minimize the biased significance of metabolites present in less abundant; the analysis allows better quantitative comparison of low MW metabolites and surmounts their discriminatory significance as well. Therefore, the lipid signals from the data matrix was excluded to discriminate the metabolic perturbations for the metabolites. Again, the residual water containing regions (δ 4.7–5.1 ppm) were excluded to avoid the imperfect water suppression. The binned data obtained from AMIX was executed by dividing each data point by the sum of all data points present in the sample, to compensate the differences in concentration of metabolites among individual serum samples.

The binned data were scaled up using unit variance where identical weight was provided to all variables. The resulting data matrices were exported into Microsoft Office Excel 2010 and used for multivariate analysis using the web-based tools server MetaboAnalyst Version 3 (http://www.metaboanalyst.ca) for statistical data modeling and analysis. Principal component analysis (PCA) was carried out on both the CMPG and sets to identify the outliers. To further demonstrate the separation between different group samples, supervised partial least squares-discriminant analysis (PLS-DA) was conducted to expose class separations between the groups and to identify the metabolites responsible for class separation. The validation of the PLS-DA models were monitored by a permutation analysis (100 times), and the resulting cross-validation parameters R^2^ and Q^2^ were used to evaluate the quality of the PLS-DA models, for example, the goodness-of-prediction parameter by Q^2^ (also referred as predictive capability of the model) and the goodness-of-fit parameter by R^2^ (also referred as explained variance). The PLS-DA model was further utilized to identify the metabolites responsible for the discrimination based on their higher values of variable significance on projection scores (i.e. VIPs) and showed the statistical significance at 0.05 level of probability i.e. p-value < 0.05 (calculated using Mann-Whitney test for pairwise comparisons). Potential metabolites markers were then extracted from PLS-DA loading plots and the VIPs. Moreover, the Student’s t-test (*p* value less than 0.05) was employed to determine the statistical significant biomarkers along with up and down regulation of metabolites.

### Statistical data analysis

Statistical data analysis was carried out using the software GraphPad Prism 5.0 (San Diago, CA, USA). The results were expressed as mean ± standard deviation (SD) (n = 8). The statistical data was analyzed by one-way ANOVA (analysis of variance) followed by Bonferroni’s multiple comparison test. Statistically significant differences were observed between carcinogen control (CC) group and test groups (PC, T1, T2 and T3). (*p < 0.001, **p < 0.01 and ***p < 0.05, when compared to the carcinogen control (CC) group).

## Electronic supplementary material

Supplementary Materials
