# Author Correction: Novel Indole-fused benzo-oxazepines (IFBOs) inhibit invasion of hepatocellular carcinoma by targeting IL-6 mediated JAK2/STAT3 oncogenic signals

**DOI:** 10.1038/s41598-020-59134-9

**Published:** 2020-02-06

**Authors:** Ashok K. Singh, Archana S. Bhadauria, Umesh Kumar, Vinit Raj, Amit Rai, Pranesh Kumar, Amit K. Keshari, Dinesh Kumar, Biswanath Maity, Sneha Nath, Anand Prakash, Sudipta Saha

**Affiliations:** 1 Department of Pharmaceutical Sciences, Babasaheb Bhimrao Ambedkar University, Vidya Vihar, Raibareli Road, Lucknow, 226025 India; 2 Faculty of Mathematical and Statistical Sciences, Shri Ramswaroop Memorial University, Deva Road, Lucknow, 225003 India; 3 Centre of Biomedical Research, SGPGIMS Campus, Raebareli Road, Lucknow, 226014 Uttar Pradesh India; 4 Department of Biotechnology, Babasaheb Bhimrao Ambedkar University, Vidya Vihar, Raibareli Road, Lucknow, 226025 India

Correction to: *Scientific Reports* 10.1038/s41598-018-24288-0, published online 12 April 2018

This Article contains errors. The Legend for Figure 6 is incomplete, as it does not specify that the control data has been published in Figure 6 of a previous study^1^; these two studies were performed simultaneously, with the same normal control, positive control, and negative control groups.

The Supplementary Information did not include full-length gel images. Uncropped images for Figure 4B are provided below as Figure 1.

**Figure 1:**
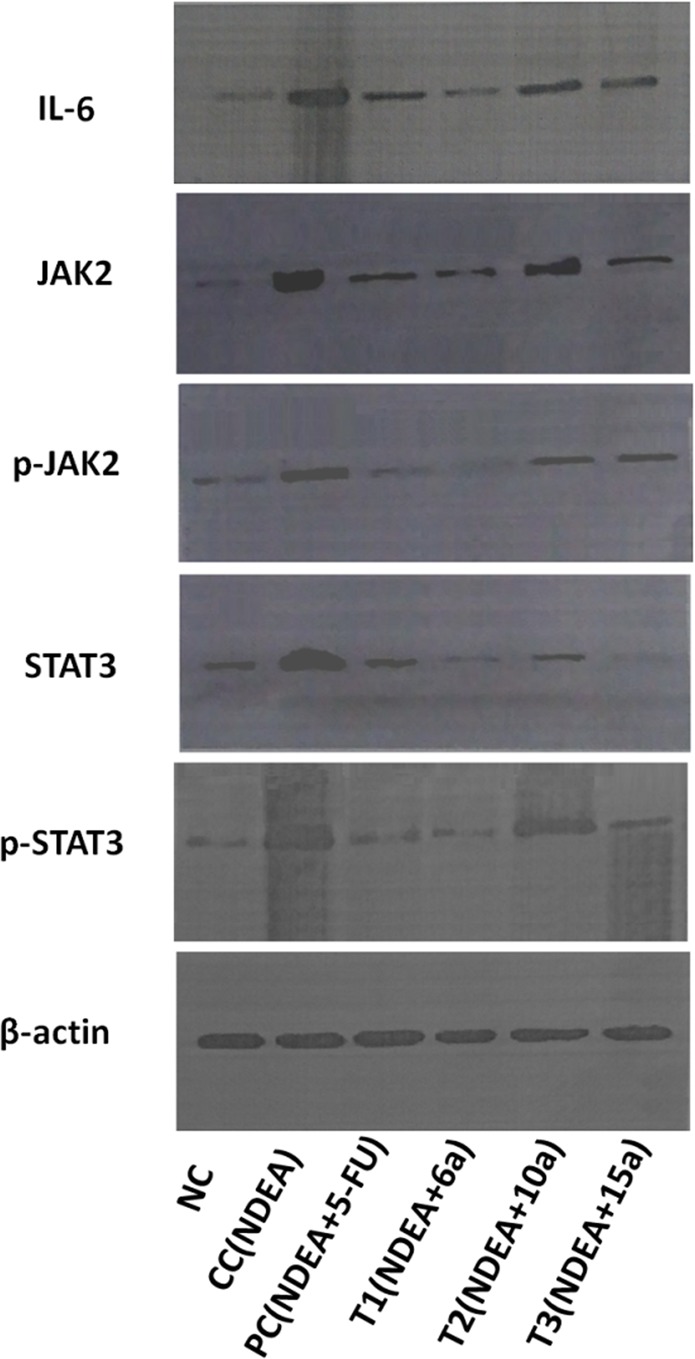
.
