# Supplementary material for: Novel Indole-fused benzo-oxazepines (IFBOs) inhibit invasion of hepatocellular carcinoma by targeting IL-6 mediated JAK2/STAT3 oncogenic signals
Source: Sci Rep. 2018 Apr 12;8:5932. doi: 10.1038/s41598-018-24288-0 (PMC5897576; doi:10.1038/s41598-018-24288-0)
Supplement: Supplementary file 1 — Supplementary Materials [file 41598_2018_24288_MOESM1_ESM.doc]

**Supplementary material**

**Novel Indole-fused benzo-oxazepines (IFBOs) inhibit invasion of hepatocellular carcinoma by targeting IL-6 mediated JAK2/STAT3 oncogenic signals**

Ashok K Singh^1^, Archana S Bhadauria^2^, Umesh Kumar^3^, Vinit Raj^1^, Amit Rai^1^, Pranesh Kumar^1^, Amit K Keshari^1^, Dinesh Kumar^3^, Biswanath Maity^3^, Sneha Nath^4^, Anand Prakash^4^, Sudipta Saha^1*^

^1^Department of Pharmaceutical Sciences, Babasaheb Bhimrao Ambedkar University, Vidya Vihar, Raibareli Road, Lucknow 226025, India

^2^Faculty of Mathematical and Statistical Sciences, Shri Ramswaroop Memorial University, Lucknow- Deva Road 225003, India

^3^Centre of Biomedical Research, SGPGIMS Campus, Raebareli Road, Lucknow 226014, Uttar Pradesh, India

^4^Department of Biotechnology, Babasaheb Bhimrao Ambedkar University, Vidya Vihar, Raibareli Road, Lucknow 226025, India

Authors for Correspondence:

Dr. Sudipta Saha

Assistant Professor

Department of Pharmaceutical Sciences,

Babasaheb Bhimrao Ambedkar University,

Vidya Vihar, Raebareli Road,

Lucknow-226025

Phone: +91-8090747008

Email: [sudiptapharm@gmail.com](mailto:sudiptapharm@gmail.com)

***Annexure I:***

**Table S1** Effects of **6a**, **10a** and **15a** on incidence of carcinogenic nodules in HCC after oral administration of 10 mg/kg for 15 days.

| **Groups** | **No. of rats bearing nodules/ total rats** | **% Incidence of nodules^$^** | **Total no. of nodules** | **No. of nodules per animal^#^** |
| --- | --- | --- | --- | --- |
| **NC** | 0/8 | 0 | 0 | 0 |
| **CC** | 8/8 | 100 | 57 | 7 |
| **PC** | 3/8 | 37.5 | 9 | 1 |
| **6a** | 3/8 | 37.5 | 10 | 1 |
| **10a** | 6/8 | 75 | 30 | 4 |
| **15a** | 4/8 | 50 | 16 | 2 |

**^$^** (Number of rats bearing nodules/total rats in each group) ×100

**^#^** (Total number of nodules/total rats in each group)

***Annexure II:***

*Serum aspartate aminotransferase (AST) and alanine aminotransferase (ALT)*

Working solutions were prepared by dissolving 4 mL of reagent 1 (mixture of L-alanine and α-ketoglutarate for ALT and mixture of L-aspartate and α-ketoglutarate for AST) and 1 mL of reagent 2 (mixture of nicotinamide adenine dineucleotide phosphate, NADP and lactate dehydrogenase, LDH) and kept at 2 to 8 °C for future use. The whole assay was performed in a cuvette and each cuvette contained 1.0 mL of working solution and 0.1 mL of plasma. This was incubated for 1 min at 37°C and the change in optical density (ΔA_340_/min) was measured per minute for the next 3 min using a UV/VIS Spectrophotometer (Labtronics, Australia). Data were calculated by the following equations^1^:

ΔA_340_/min = [A_340_ (time 2) - A_340_ (time 1)]/[time 2 (min) - time 1 (min)]

ALT or AST Activity (U mL^-1^) = ΔA_340_/min × 1746 × 10^-3^

*Plasma alkaline phosphatase (ALP)*

All groups contained 1 mL buffer substrate and 3 mL distilled water. Later, 0.1 mL distilled water, 0.1 mL phenol and 0.1 mL serum were added to the blank, standard and test groups, respectively. All the groups, including the normal control, were incubated for 15 min at 37°C. Then, 0.1 mL of plasma was added to the control group after incubation. All the tubes were mixed properly and absorbance was measured at 510 nm of wavelength.

Plasma ALP was calculated as follows^1^:

ALP activity (U mL^-1^) = [A (test) - A (control)/A (standard) - A (blank)] × 7.1 × 10^-3^

*Tissue thiobarbituric acid reactive substances (TBARS)*

TBARS assay was performed as per the method prescribed in the previous literature with slight modifications^2^. 1.0 mL of 10% (w/v) tissue homogenate, 0.5 mL of 30% trichloroacetic acid and 0.5 mL of 0.8% thiobarbituric acid were taken together in a falcon tube and covered with aluminium foil. Then, the tubes were kept in a shaking water bath for 30 min at 80ᵒC. Later, it was cooled for 15 min and centrifuged at 3000 rpm for 15 min. Absorbance was recorded spectrophotometrically at 540 nM against blank in which tissue sample is absent. The amount of MDA present in a sample was calculated according to the following equation:

nM of MDA/µg of protein = (V × OD at 540 nM) / (0.56 × protein concentration),where, V is final volume of the test solution.

*Tissue protein carbonyl (PC)*

PC assay was performed as per the method prescribed in the previous literature with slight modifications^2^. 10% tissue homogenate was prepared in distilled water. 150 µL of tissue homogenate was taken in eppendorf tube and precipitated by adding 500 µL of 10% trichloracetic acid. Then the tubes were centrifuges at 13,000 rpm for 2 min and supernatant was discarded. Later, the cell pellets were incubated with 500µL of 0.2% 2,4-dinitrophenylhydrazine with constant vortexing at every 5 min interval for 1 h. After that, supernatant was removed and cell pellets were washed with 500µL ethanol:ethyl acetate (1:1) solution three times. At last, pellets were dissolved in 600µL Guanidine Hydrochloride (6M) and absorbance was measured at 360 nM. Blanks solution was prepared in the similar procedure where cells were absent. The PC content was calculated as follows:

PC (µg/mg of protein) = (A360 sample – A360 sample blank)/mg of protein

*Tissue glutathione (GSH)*

GSH assay was performed as per the method prescribed in the previous literature with slight modifications^2^. 0.2 mL of 10% (w/v) tissue homogenate was taken in eppendorf tube and 1.8 mL distilled water added to it. Simultaneously, we prepared precipitating solution by mixing 1.67 g of glacial metaphosphoric acid, 0.2 g ethylenediaminetetraacetic acid disodium salt and 30 g sodium chloride in 100 mL distilled water. This precipitating solution was added to the above mixture. The mixture was then allowed to stand for 5 min and filtered. To 2 ml of filtrate, 1.0 mL of 0.4% w/v 5,5’-dithio-bis-2-nitrobenzoic acid and 8.0 mL of 0.3 M phosphate solution were added and centrifuged at 13000 rpm for 1 min. A blank was prepared in the similar procedure where tissue sample was absent. Then, the optical density (OD) was measured at 412 nM. The total protein content of each sample was measured using the Bradford reagent and bovine serum albumin (BSA) was used as a standard.The tissue GSH content was calculated as follows:

GSH (μM/µg of protein) = (310.4 × Ei× OD at 412 nm)/µg of protein, where Ei is the correction factor (0.542).

*Tissue superoxide dismutase (SOD)*

Determination of SOD in the test samples were performed as per the method prescribed in the previous literature with slight modifications^2^. 100 µL of 10% cytosolic supernatant was prepared with tris–hydrochloric acid buffer (pH=8.5) and final volume was adjusted up to 3.0 mL with the same buffer. Finally, 25µL of pyrogallol was added and change in absorbance was recorded at 420 nM at one minute interval for 3 minutes. Blank was prepared in which tissue sample was absent. One unit of SOD is described as the amount of enzyme required causing 50%inhibition of pyrogallol auto-oxidation per 3 mL of assay mixture and is given by the formula:

Unit of SOD / µg or protein = [100 × [(A-B)/ (A×50)]]/µg of protein

where A = Change inabsorbance per minute in control and B = Change in absorbance per minute in test sample.

*Tissue catalase (CAT)*

CAT enzyme estimation was performed as per procedure which was described previously^2^. 10% (w/v) tissue homogenate was prepared in 50mM phosphate buffer and centrifuged at 10000 rpm for 20 minutes. 50 μL of supernatant was added to a tube containing 2.95 mL of 19 mM solution of hydrogen peroxide (H_2_O_2_) prepared in potassium phosphate buffer. Disappearance of H_2_O_2_ was monitored at 1 min interval for 3 mins at 240 nM. CAT activity was calculated as follows:

nM of H_2_O_2_/min/µg of protein = (ΔA/min × volume of assay) / (0.0719 × volume of sample × µg of protein)

***Annexure III:***

*Tissue bilirubin*

Bilirubin in liver was measured as per the following procedure published earlier in the literature with slight modifications^2^. All the tissue samples were thawed and homogenized in phosphate buffer saline (8.0 g sodium chloride, 0.2 g potassium chloride, 0.2 g potassium dihydrogen phosphate, 1.15 g disodium hydrogen phosphate, 0.372 g ethylenediaminetetraacetic acid disodium salt, pH 7.4). 500 µL of tissue homogenate (10%) was added to 2.0 mL of 1.5% butylatedhydroxy toluene in acetone:ethanol (1:1) in a eppendorf tube. Simultaneously, fresh diazo reagent was prepared by mixing 300µL of 10% sodium nitriteand 8.0 mL of 2M p-toluene sulfonic acid, then combining 4.0 mL of this mixture with 2.0 mL of 2.1% p-iodoaniline in glacial acetic acid, kept at room temperature for 2.0 min. Then this solution was diluted with distilled water (10 mL) and 200µL of 1.5M ammonium sulfamate. This working diazo reagent was kept on ice for 5min and 500µL was added to each sample homogenates. Diazo blank reagent was freshly prepared by combining 2 mL of p-toluene sulfonic acid and 5.0 mL of 10% ascorbic acid, followed by addition of 2.1% *p*-iodoaniline in glacial acetic acid and 2.0 mL of n-butyl acetate, mixed and used immediately. Finally, all the tubes were incubated for 1 hour on ice in dark. After incubation freshly prepared 3.0 mL of 1% ascorbic acid in 0.1 M sodium chloride was added to each vial. All the vials were shaken vigorously, kept for 1.0 min and centrifuged at 2400 rpm for 10 min. The absorbance of the upper organic phase was taken at 530 nM wavelength. The content of bilirubin was calculated as follows:

A530sample – A530 sample blank = ΔA530Test

*Tissue biliverdin*

Estimation of biliverdin was performed as per the method prescribed in the previous literature with slight modifications^2^. Tissue samples were homogenized in phosphate buffer saline as per described in the previous section. 500 µL of tissue homogenate (10%) was combined with 500µL of 10 M glacial acetic acid, 400µL of 40 mM ascorbic acid, 500µL of double distilled water and 100µL of 200 mMbarbituric acid. Samples were incubated in a water bath at 95°C in dark and then samples were extracted with butanol, vortexed and centrifuged. The upper organic layer was carefully removed and extracted with 2.5 mL 2M sodium hydroxide. The absorbance of upper layer was taken at 535 nM wavelength. The content of biliverdin was calculated as follows:

A535sample – A535 sample blank = ΔA535Test

***Annexure IV:***

*Histopathology and SEM analyses*

Histopathological studies were also performed to find out the morphological changes of liver cells after IFBOs administration. Liver tissues from each group were assessed for their morphological changes using hematoxylin and eosin staining. The tissues were preserved in 10% formalin overnight. Next day, the cells again were superseded by 70% isopropanol overnight. Later, the tissues were exposed to isopropanol at various concentrations (70, 90, and 100%) and dehydrated by 100% xylene. The tissue samples were then embedded in bees wax and 5 µM sections were prepared by using microtome. Then, the tissues were succeeded by hematoxylin and eosin staining and observed under microscope (magnification 40X)^2^.

For SEM analysis, liver tissue samples were collected (2–4 mm) and fixed in 2.5% glutaraldehyde for 2–6 h at 4°C for primary fixation. Then, the samples were washed with 0.1 M phosphate buffer for 15 min at 4°C. After that, 1% osmium tetroxide was used as a post-fixation for 2 h at 4°C. Again, the samples were washed in 0.1 M phosphate buffer for three times at 15-min interval and kept at 4°C. Later, these samples were dehydrated with acetone at various concentrations (30%, 50%, 70%, 90%, 95%, and 100%). After that, all specimens were air dried at room temperature and critical point drying (31.5°C at 1100 psi). Finally, samples were mounted on to the aluminum stubs with adhesive tape and observed for the morphological changes using SEM (JEOL JSM-6490LV)^2^.

*References*

1. Kushwaha PS, Raj V, Singh AK, et al. Antidiabetic effects of isolated sterols from *Ficus* *racemosa* leaves. RSC Adv. 2015;5:35230-35237.
2. Rawat A, Chaturvedi S, Singh AK, Guleria A, Dubey D, Keshari AK, Raj V, Rai A, Prakash A, Kumar U, Kumar D, Saha S. Metabolomics approach discriminates toxicity index of pyrazinamide and its metabolic products, pyrazinoic acid and 5-hydroxy pyrazinoic acid. Hum Exp Toxicol. 2017. doi: 10.1177/0960327117705426.
